# Food fraud data collection needs survey

**DOI:** 10.1038/s41538-019-0036-x

**Published:** 2019-05-16

**Authors:** John Spink, Christopher Elliott, Moira Dean, Cheri Speier-Pero

**Affiliations:** 1 College of Veterinary Medicine, Michigan State University, 1129 Farm Lane, East Lansing, MI USA; 2 Institute for Global Food Security, Queen’s University Belfast, University Road, Belfast, BT7 1NN Northern Ireland UK; 3 Institute for Global Food Security, Queen’s University Belfast, University Road, Belfast, BT7 1NN Northern Ireland UK; 4 Department of Supply Chain Management, Broad Business School, Michigan State University, 632 Bogue St. N520, East Lansing, MI 48824 USA

**Keywords:** Economics, Science, technology and society

## Abstract

This research project was conducted to understand the data collection needs when addressing food fraud prevention. The foundation for an understanding of the fraud opportunity utilizes a holistic and all-encompassing information sharing system. The anonymous online survey was distributed first to a targeted group of food fraud leaders from manufacturer or brand owner companies and then to a public group. From the 96 survey responses, first, regarding “data” there is generally “enough” and “good enough” data to meet the current assessments and compliance needs. Second, regarding the process, there is a need for more guidance or harmonization on vulnerability assessments, strategy development and management, and correlation to all other enterprise-wide risks (ERM/COSO). Third, there is the general activity of conducting food fraud vulnerability assessments, but there is a lack of clarity or direction on the scope (all types of fraud) and confidence in the conclusions (a clear insight or diagnosis of root-cause). This survey suggests there is a need for more definition and formality of the method and process for addressing food fraud. Finally, a focus on harmonizing terms, vulnerability assessment methods, and then of common policy/strategy will enable the risk assessors to define their future data collection requirements and needs. Further research should explore the specifics of the data collection needs and expand to other stakeholders such as regulators and enforcement.

## Introduction

Food fraud is a recent topic for research and regulatory action. A series of incidents have raised the public and industry focus on assessing the problem and selecting countermeasures and control systems. One major preliminary step is to review incidents and to conduct initial vulnerability assessments. Understanding the types of incidents is essential for the industry as well as the regulators and enforcement. Data collection is a key component to those preliminary steps.

This research project was conducted under a subcontract to Michigan State University’s Food Fraud Initiative (MSU FFI) from Queen’s University Belfast for the UK Economic and Social Research Council (ESRC) on “Analyses of food supply chains for risks and resilience to food fraud/crime” ESRC Grant relating to Experts and Data Collection/Information Sharing System.” The research justification for this effort is that food fraud related emerging vulnerability assessments are being conducted and included evolving data needs.

Food fraud is an illegal deception for economic gain using food. ^1–15^ A subcategory of food fraud is economically motivated adulteration (EMA) which is defined by the US FDA working definition as a “substance” (e.g., adulterant-substance) for “economic gain” with a “health hazard.” While there are several lists, the general types of food fraud include adulterant-substances, tampering, theft, stolen goods/production over-runs, smuggling, gray market/diversion, and intellectual property rights counterfeiting.^1^ As the concept is getting more government and industry scrutiny, there are new methods and processes to define, assess, implement, and monitor the vulnerabilities.^16–20^ These emerging concepts define a requirement for new and specific data sets.

Currently, regulations and certifications require “a” process to assess and manage hazards but do not prescribe a specific approach or method.^3,21,22^ The general food safety, food quality, or consumer protection laws—assuming the incident was not intentional there is an application of strict liability (e.g., responsibility regardless of negligence or intent) or vicarious liability (e.g., responsibility for a colleague, employee, or accomplice)—require a company to manage and control their products. For example, the U.S. Food Drug & Cosmetics Act of 1938 was updated by the U.S. Food Safety Modernization Act of 2011 to include: “The hazard analysis must be written, regardless of the results of the analysis, and must include two elements: (1) a hazard identification (hazard assessment) and (2) a hazard evaluation (control plan).” While the two steps are clear for food related health hazards, for food fraud, there are no further details or specifications. Due to the requirement of “a” process but little additional details, industry groups and standards organizations such as GFSI, ISO, Codex, and INFOSAN have responded by further reviewing the problem.

The compliance and certification requirements are only now starting to be defined. An example of lack of a prescribed approach is evident in the industry Global Food Safety Initiative (GFSI) requirements that were published in February 2017 and were a compliance requirement a year later in January 2018.^3^ For GFSI compliance, addressing food fraud is not optional and requires (1) “a” vulnerability assessment only for food fraud and (2) “a” prevention plan for the hazards identified.^3^ The GFSI requirements are defined in standards created by Certification Programs Owners (CPO or CP), formerly referred to as “Scheme Owners”). The standards are judged by third-party Certification Bodies (CBs). To help the industry with compliance, GFSI has endorsed the SSAFE Organization’s Food Fraud Guidance that includes a Food Fraud Vulnerability Assessment (FFVA). The publically available and free SSAFE FFVA covers most—but not all—types of food fraud. Other publically available and free models include the US Pharmacopeia Food Fraud Mitigation Guide which covers adulterant-substances in ingredients and the Food Fraud Initials Screening tool (FFIS) which covers all types of fraud and is based on COSO Enterprise Risk Management.^19,23,24^

When considering the data collection needs, it is essential to:

- Address all types of food fraud not just adulterant-substances: GFSI (and regulations including the U.S. Food Safety Modernization Act Preventive Controls Final Rule) require addressing all “agents” that lead to a possible or actual “health hazard” that are “economically motivated” so the compliance requirements will be for all types of food fraud.^25^
- Conduct a FFVA for all types of fraud, but level of detail in undefined: The current regulatory and certification requirements are only defined to conduct “a” vulnerability assessment with little additional specificity.
- Consider other enterprise-wide financial or securities assessments that are a non-food compliance or certification requirement: These are industry-specific financial reporting requirements such as Sarbanes-Oxley in the USA and others in other countries or regions. These regulatory compliance requirements include statements of internal controls and an integrated framework for reporting of risks to equity such as crime or fraud. Non-compliance can lead to fines or criminal penalties for the corporate leaders including the Board of Directors.
- Conduct a first FFVA and create a food fraud prevention strategy (FFPS): There is a need to first create the system and structure before conducting advanced analysis and decisions on complex countermeasures or control systems. The resource-allocation decision-maker will define the level of detail required in the FFVA to make a decision.
- Consider future needs will be specified to support specific decisions: Once the base assessments or strategies are implemented the more detailed data or assessment needs can be defined.

Before framing the exact data collection needs, it is essential to review the types of assessments or analysis that may be conducted. The previous reports and publications have often been broadly presented as a summary or overview of the problem and not yet expanded to consider the specifications of data science. One approach is to think about the analysis as a “data analytics” or “big data” project. The specific characteristics of data and the type of problem addressed will influence the specific data analytics technique that should be applied. Finally, a primary application of analytics is using Intelligence Analysis by the Intelligence Community or the food safety researcher. This set of considerations provides an important foundation for the data collection needs survey method development.

Before reviewing the survey, it is important to more fully discuss the science of data analytics or commonly referred to as “Big Data.” The specifically an important consideration is of the characteristics of the data. Data analytics involves the application of a broad array of analytical techniques to data that might be available to an organization, supply chain partners, a government agency, or any other entity that has a repository of data. While there are a growing set of techniques that can be implemented, data is perhaps the most critical driver in the emergence of data analytics. Changes in technology and business practice have created greater volumes and type of data that facilitate more analysis that can lead to a more accurate prediction of future outcomes. The emerging science of “Data Analytics” identifies specific characteristics of data sets varying from the “3 V’s” to the “7 V’s”.^26–31^

The broadest characterization of data and why this data drives opportunities for predictive modeling comes from the US National Institute of Standards and Technology NIST:^28^

1. Value, refers to the inherent actionable decision making that drives economic contributions to the organization when analyzing data. Value creation can occur at many levels including strategic (e.g., developing an early identification analytical model of food fraud within an organization, supply chain, or industry), operational (e.g., implementing an analytical model that compares raw material vendors on a variety of characteristics including organizational, supply chain, or industry data on the raw material purity/adulterant issues; and tactical (building an analytical model that can track on a real-time basis any contaminants or food particle mixes that exceed specific levels.
2. Variability, refers to the changes that may occur in the data over a period. These changes may reflect inherent variability in the underlying composition to a food product due to changes in underlying seed composition, fertilizers, etc. that could affect the manner in which food products are assessed for being within tolerance or possibly exhibiting adulteration.
3. Variety, refers to data breadth of data types available to organizations in today’s business environment. While traditional “structured” data (e.g., the transactional data in an organization’s database) is the primary source of data used in a predictive analytical model, there many other data types. For example, organizations can “scrape’ data from Facebook, Twitter, etc. to incorporate unstructured text-based data into an analysis. Further, sensor data can be invaluable to identify potential quality issues with food products in transit. For example, “Internet of Things (“IoT” types of sensors allow both food manufacturers and logistics providers to track the temperature, humidity, and precise location of food items during all transit to facilitate monitoring and quality issues.
4. Velocity, refers to the rate of data flow and is often evaluated as the real-time nature of data flow. If it is important for an organization to know exactly where a product has been, the IoT sensors can facilitate shipment tracking (e.g., variety). If this data were monitored real-time, the data flow would increase the velocity of the data, creating challenges in capturing, storing, and ultimately, analyzing the data.
5. Veracity, refers to the inherent “truthfulness” of the data. Truth reflects the underlying accuracy of the data and cleansing the data to remove erroneous or missing data is a critical and typically very time-consuming activity in any data analytics undertaking. In addition, veracity focuses on the degree to which the data appropriately captures the meaning the data scientist ascribes to the behavior or outcome associated with the data (e.g., if analyzing employee time spent on an activity then is “time” actually a measure of productive activity or might it reflect the time an employee has spent using a computer for non-work activity [e.g., social media]).
6. Volatility, refers to the tendency for data structures to change over time. For example, an organization may have a system that monitors contaminants that involves taking a sample of each incoming shipment, testing the food product for its underlying chemical composition, and updating a database with the results for each sample tested. If a technological innovation were to facilitate real-time analysis of the entire shipment, the data structure (what is stored in the database and how to interpret the data) would change.
7. Volume, refers to the size of the dataset. Not surprisingly, most organizations are generating and storing more data (by many orders of magnitude) than they have even in the most recent past.

Since data veracity is such an important concept more detail is provided:^28^

Veracity refers to the completeness and accuracy of the data and relates to the vernacular “garbage-in, garbage-out” description for data quality issues in existence for a long time. If the analytics are causal, then the quality of every data element is extremely important. If the analytics are correlations or trending over massive volume datasets, then individual bad elements could be lost in the overall counts, and the trend will still be accurate. [Many] people debate whether “more data is superior to better algorithms”, but that is a topic better discussed elsewhere.

As organizations begin the process of identifying the high priority analytics projects, a first organizational step is to assess what data is generated or accessible and whether or not it is currently stored. This data should then be “cleansed” (often the most time demanding process in any analytics project) so that a formal analysis and predictive model can be developed. For the data collection needs, it is important to consider the source and type of information before determining the type of analysis that will be conducted.

Next, another important aspect of data analytics or big data is the type of analysis. There is tremendous diversity in the analytical techniques that an organization can use to make sense of its data. The first step is to evaluate the goal associated with an analytics activity more carefully, and there are three types of decision-making goal outcomes:^29^

- Descriptive analytics: Such techniques focus on highlighting relationships amongst the data from a specific dataset. The evaluation is of the specific dataset and may or may not be representative of the entire market. For example, descriptive analytics apply simple and more complex statistical technique to describe (e.g., correlations, probabilities, etc.) what is contained in a dataset or database. This analysis can lead to the identification of factors that might be causal that would be evaluated in a predictive analysis to drive desired outcomes (e.g., identifying specific adulterant-substances).
- Predictive analytics: Applying statistical and other types of machine learning techniques to predict the likelihood of future occurrences. Predictive models often involve analyzing a subset of the data of interest to “train” a predictive model. This training may involve linear regression or other techniques that enable the data scientist to discern which factors drive an outcome of interest and the relative importance or weighting associated with that factor. The data scientist then “tests” the model against the remainder of the data to ascertain the predictive accuracy of the model (e.g., if the trained model predicts an outcome 70% of the time, does it predict the same outcome in the test data at the same rate?). If the predictive accuracy is strong, the organization can then develop operational strategies for driving increases or decreases to the key drivers to improve the outcome of interest. Of importance is that the organization continues to capture and analyze data to know if these operational strategies improve the outcome score. Also, the organization should re-evaluate its predictive model as the key drivers might have changed due to the changes in operational activities and how stakeholders received them. “Advanced statistical, information software, or operations research methods to identify predictive variables and build predictive models to identify trends and relationships not readily observed in a descriptive analysis.” One use is “to build predictive models designed to identify and predict future trends (e.g., ANOVA and multiple regression analysis).”
- Prescriptive analytics: Finally, this assessment leverages the predictive analytics assessments and guide decision makers regarding the “best” course of action. Such analytics often involve techniques focused on optimization (e.g., linear programming) to identify the best combination of resources to achieve a specific outcome. The application of this analysis would create systems where a response is automatic such as modifying a manufacturing process.

Once the nature of the data and the type of analysis is determined, it is efficient to review a specific application to food fraud prevention. One use is intelligence analysis, which is a specific field of Criminology study that has methods and procedures.

For the application of the data analytics or big date, there is an important consideration of the intelligence analysis evaluation and methods. Before converting a piece of raw data to information and then to actionable intelligence, it is important to apply intelligence analysis methods to evaluate the incoming information by (1) source and (2) type of information. While there are variations, a typical “4 by 4” method is published by the United Nations “Guidance on the preparation and use of serious and organized crime threat assessments (SOCTA)” section on “A method for evaluating information,” “the most commonly applied and understood is the 4 × 4 (four by four) system”.^32^ It is beyond the scope of this project but the 4 × 4 system is often adapted for specific purposes such as the “3 × 5 × 2” assessment. What is common is that there is specific, harmonized, and standardized method and system to judge the source and type of information. The method presents four levels of “Sources” considered with four levels regarding the “Type of information”. The reports stated that “When communicating the intelligence analysis there should be sufficient detail to allow the reader to assess the validity of the source” (Table 1).

**Table 1:** Conventions for evaluating the source and type of information (UNODC 2010)

Factor	Source	Factor	Information
A	Where there is no doubt of the authenticity, trustworthiness and competence of the source, or if the information is supplied by a source who, in the past, has provided to be reliable in all instances	1	This is information where the accuracy is not in doubt
B	Sources from whom information received has in most instances proved to be reliable	2	This is information which is known personally to the source but not known personally by the official passing it on
C	Sources from whom information received has in most instances proved to be unreliable	3	This is information not known personally to the source but corroborated by other information already recorded
X	The reliability of the source cannot be assessed	4	This is information which is not known personally to the source and cannot be corroborated

For example, a “4 by 4” assessment of information may result in a range from a high of “A1” to a low of “X4.” While usually new for food scientists, this type of ranking system is commonly understood in the Intelligence Community. For the data collection needs, it is essential to consider all the characteristics and information evaluation. The characteristics determine the type of analysis that can be conducted.

For the risk assessors applying data analytics or big data, there is a very fundamental and preliminary task of framing the question. Except where risk assessors “just start gathering raw data”, many organizations struggle with how to initiate an analytics project. Questions include: what problems should be examined? Do we have the “right” data? Our experience, which has been supported in practice, is that an organization can begin with any question and any data and build their capabilities and the resulting value of the analytics activity.

Thus, when evaluating the application of analytics to food fraud, each organization should be able to identify the most salient risks to its specific products and processes. Further, based on those risks, the organization can determine what data is most critical to evaluate and over what period (and what frequency (e.g., daily, hourly, real-time) to evaluate potential food fraud vulnerabilities.

When such an assessment is made, the organization may determine that the type (e.g., variety), or amount (e.g., the volume of data over time across business partners, etc.) of data is not as robust as is required for analysis. The organization can then put processes in place to capture and store this data.

For food fraud, there are a range of activities that require data for assessment. There are food fraud databases, but each have a wide range of goals, so none were intended to be comprehensive. Specific databases were developed to meet specific needs. There are a range of needs for data in the field of food fraud:^19,33,34^

- Overview of the problem—General
- Overview of the problem—Detailed
- Negative list/Black list (including “Early Warning System” for known concerns)
- Food Fraud Vulnerability Assessment—Current state
- Food Fraud Vulnerability Assessment—Fraud opportunity
- Product fraud incident clustering (general review of data sets)
- Ongoing suspicious activity scouting/Horizon scanning
- Criminal prosecution—use as evidence during an investigation or court case

Food fraud has been ongoing since the beginning of commerce but has only recently become a major regulatory and industry focus. The UK DEFRA Elliott Review interim report was published in 2013 which began the UK focus on the subject.^2^ The UK created the National Food Crime Unit (NFCU). Additional funding was allocated by the UK by Economic and Social Research Council (ESRC) on “Analyses of food supply chains for risks and resilience to food fraud/crime” (note: the funder of this research, see acknowledgments). Also, the US Food Safety Modernization Act was published in 2011 but enacted through the Preventive Controls Rule in July 2016. After the horsemeat incident in Europe, the European Commission funded USD$14 million for the Food Integrity Project. The US Food and Drug Administration (FDA) has reinforced that “all” types of food fraud—or more specifically their term of “economically motivated adulteration” or “EMA”—has been illegal since at least the Food Drug & Cosmetics act of 1938 (FDCA). In 2018, Codex Alimentarius (CODEX) formed an Electronic Work Group (EWG) to create a discussion paper on the definitions of terms related to food fraud as well as a gap analysis for the standards. World Health Organization (WHO) and Food and Agriculture Organization of the United Nations (FAO) manage the International Network of Food Safety Authorities (INFOSAN). INFOSAN conducted a member survey on food fraud concerns and needs.^35^ INTERPOL-Europol has just launched their 7th Operation Opson which focuses on specific food crimes that are fraudulent or counterfeit.^36^ The European Commission created a Food Fraud Network for enforcement partners and has continued to fund several initiatives such as the Food Integrity Project and now a Horizon 2020 joint project between the EU and China. As mentioned the industry or commercial standards have been defined and are now required as of January 2018.

While this regulatory and compliance requirement activity has been ongoing, companies and enforcement agencies have been developing and implementing food fraud prevention related projects. The data collection and needs are currently being defined and redefined. This research project was funded in 2014 to contribute to understanding those needs.

## Results

The survey was conducted from July 2015 to September 2016 and resulted in 96 responses. The responses were separated by different mailings and then compiled into the final sample. The “core expert group” responses were compared to the general “public” group, and major differences were noted below. The two groups were compared to evaluate any differences that could be due to speculation or uniformed bias. Based on the nature of the survey items and respondents, descriptive statistics were applied. The discussion of the results will be separated by research questions. There are six thematic areas noted below.

### Demographics

This first set of demographic questions is a standard MSU Food Fraud Initiative set that allows for meta-analysis across research projects. This “high-involvement” survey population covered a broad range of perspectives on the research questions. The survey population was 81% from “Food Producer/Brand Owner/Distributor” category and 7% from “Suppliers or Contractors,” and the rest across government/consultant/academia/other. The industry groups were 70% from “Manufacturing” and 11% from “Retailers” while 81% were from “Headquarters” compared to 9% from “plant/local facility.” The titles were 22% Vice-President, 20% Director, and 39% Manager. Of those, 41% had been at their company and in their positions 5–14 years. The companies were 40% from <USD$ 1 billion revenue companies and 18% from <USD$20 million, with 38% over 1,000 employees and 10% less than 100. For operations, 60% were global, and 82% described their “supply base” as global (see table for full results). The most important detail applied to this survey is that 91% stated they do address food fraud issues in their current position. The findings were that regardless of the company size, this was an expert population who are familiar with food fraud prevention.

### Food fraud management

The scope of “food fraud” is important to define to understand the data collection needs of how they used data and what additional methods or products are needed.

The respondents stated their food fraud scope is primarily—but not exclusively—on adulterant-substances (79%) and then a drop-off to second set of tampering (41%), theft (31%), smuggling (18%), gray market/diversion (26%), and counterfeiting (29%) (Table 2).

**Table 2:** Food fraud management—The scope of food fraud prevention activities

Q14: What does your company consider to be the scope of your Food Fraud activities or prevention program? (note all that apply):						
	Adulterant-substances	Tampering	Theft	Smuggling	Gray market/diversion	Counterfeiting/intellectual property rights
N ttl	69	50	37	18	22	29
% ttl	79	41	31	18	26	29

Also, the current efforts were mainly on ingredients (43%, e.g., incoming goods or raw materials) and finished goods (13%, e.g., outgoing goods or packaged products), and some did cover all types that are a compliance requirement for GFSI and FSMA (20%). Generally, companies did conduct FFVAs by “groups of products and for some individual products” (28%), by “individual items for all products and suppliers” (24%) and “only by groups of products” (22%). The magnitude of the effort can be reviewed by considering that a billion dollar revenue manufacturer could have 1000 suppliers who each provide 10–30 products, which would equate to 10,000–30,000 individual FFVAs.

A key finding is that the current focus by companies is on food ingredients and adulterant-substances. This focus will need to expand to meet regulatory and certification compliance such as FSMA and GFSI.

For the FFVA, most respondents have conducted “at least one” vulnerability assessment (52%) and most of the rest will come in the next year (27%) (Table 3). For the FFVA, most adapt someone else’s model (41%) or entirely use another’s (18%), and 13% developed a unique FFVA (28% had not conducted the FFVA yet).

**Table 3:** Food fraud management—frequency and type of assessment

Q20 How frequently will your company conduct, schedule or update your FFVA? (All responses consider that there may be occasional emergencies where your company might need to do a quick, emergency assessment.)						
	One time	Annually	Quarterly	Monthly	More than Monthly	Never
N ttl	5	49	9	1	0	12
%ttl	9	42	11	0	0	7
Q21: Was the nature of the “data” used in your FFVA:						
	Qualitative	Semi-Quantitative	Quantitative	Don’t know	Prefer not to answer	Not applicable, we did not conduct an FFVA
N ttl	24	20	2	6	1	23
%ttl	26	43	4	3	0	15

A key finding is that many companies have not defined their exact data, information, or intelligence analysis needs since they have not yet even conducted an FFVA. Even fewer companies have conducted GFSI or FSMA compliant FFVAs for their entire company.

Regarding the data to conduct the FFVA, most respondents found “enough data” (55%), and some did not (10%) with another group who did not know (28%). To address the velocity of data collection the most important finding is that the vast majority of companies currently plan to conduct their FFVAs meeting some level of GFSI compliance annually (42%), quarterly (11%), but no updated assessment monthly or more than monthly (0%). There were 9% who planned only to conduct the FFVA once.

A key finding regarding data needs is that companies are conducting FFVAs and they are generally finding “enough data” for these initial exercises. It appears they have not completed FFVAs for their entire product line, and they will continue—or be required to continue—expanding their system but may not need more data for these initial screening or pre-filter assessments.

A key finding regarding model development is that companies are utilizing published FFVA models either directly or with modification. This insight indicates there is a need for a more holistic overall prevention strategy model.

A key finding regarding data velocity is that to conduct their annual, or quarterly FFVAs companies do not need rapid access to data. That said there might eventually be “early warning systems” or other “suspicious activity reporting” systems to serve crisis management needs. At this point, those systems are not developed or implemented, so it is logical there is no current expressed need for more data. Note: The GFSI (e.g., BRC, FSSC, SQF, IFS, etc.) requirement states a requirement for at least an annual FFVA update.

For the FFVAs, a major type of data used was semi-quantitative (43%), and then qualitative (26%) followed by quantitative (4%). This insight could be due to the nature of the compliance requirement, or other initial FFVAs focused on those broad assessments of the food fraud vulnerability (e.g., initial screening or pre-filter versus the detailed assessments). In addition, companies used a wide range of sources of data including food fraud databases (61%), open source non-food fraud databases such as recalls (50%), support from outside consultants but not necessarily data sets (15%), open internet searches (44%), company internal sources (39%), developed proprietary databases (13%), and finally commercial (paid) database (7%). It is important to note that the USP Food Fraud Database was one of the most used free resources and as of September 2017 it is now a subscription, fee-based system. Since the survey was conducted there have been new fee-based databases and some of the free databases are not fee-based. This could change the findings and will be addressed in future surveys.

A key finding regarding qualitative versus quantitative data is that it was interesting was that so few respondents needed quantitative data for their current needs. This seemed to contrary to a public focus on analytical and quantitative assessments. This insight could be due to the nature of the initial food fraud vulnerability assessments as compared to future more precise “risk-based” decision-making systems (e.g., from descriptive advancing to predictive or prescriptive analytics). The respondents did not express a need for more precise, accurate, or certain food fraud data.

### Data availability and needs

This section will expand on how companies make assessments, the initial data needs and to consider the future needs for more data.

Three questions addressed if companies pay for “more” or “better” data. This question did not define how much the companies would pay or when (e.g., farther in the future to support the next iteration of more detailed vulnerability assessments). The main insight was that for “incident or seizure data that was statistically significant or determined to be of high quality, do you think your company would pay for the information?” The “core expert group” survey populations stated that while the “accuracy/precision/certainty” of the data was undefined, the majority would pay for more data (61%, if the data more thoroughly met their needs), many did not know (33%), few explicitly stated they would not (11%). This question was used in the pilot survey to the “Core Expert Group” but not included in the full survey since is shifted focus from the data collection to commercial questions.

A key finding regarding the data need is that companies will pay for more data if it meets their needs. Their needs are undefined including specification of accuracy, precision, and certainty, or if the database had to be all-inclusive of all food fraud data needs. The next step will be to define and implement the assessment to specify the exact data needs. Earlier questions addressed timeliness identifying that the FFVAs were conducted annually or quarterly.

The next three questions addressed how companies would use the results of the FFVAs. The question set was included to understand if the need was to support decisions that were “risk-informed” (e.g., the assessment provides insight for a decision) or “risk-based” (e.g., the assessment prescribes a decision). Generally the major use of the FFVA (similar concepts are combined) will be to create and implement a food fraud prevention strategy (80%), prioritizing and selecting countermeasure programs (68%), to determine brand protection needs (56%), and to adapt or improve supplier contracts (58%) (Table 4). Of those questions, few felt their “method for conducting your FFVA is “robust enough” to make these decisions” (referring to the questions in the previous sentence, 77%, 66%, 38%, and 44%, respectively). Of those responses stated they had enough data for a “risk-based” decision (referring to the same list, 71%, 55%, 33%, 49%, respectively).

**Table 4:** Data availability and needs

Q23: What decisions will your company’s completed FFVA support (check ALL that apply):		
	N ttl	% ttl
Create and implement a food fraud protection strategy (FFPS)	46	80
Selecting countermeasures or systems to combat food fraud	36	68
Add/reduce the assigned workforce	10	27
Crisis management/Initiate a crisis management team action	17	25
Initiate a product recall/stop shipment	21	31
Conduct additional investigation/authenticity tests	34	60
Initiate a commercial or criminal investigation (contact government)	5	12
Cancel a supplier contract	26	46
To adapt detail of, or improve supplier contracts	31	52
Add a product or supplier to a “blacklist” of forbidden products (cancel or ban a supplier from selling to you)	19	36
To determine brand protection risks and needs	28	56
To assess insurance needs or to report to insurance providers	9	21
Other	1	0
None	11	6

A key finding regarding data needs is that the major focus of the FFVAs was to make qualitative or preliminary decisions such as setting-up and implementing FFPS programs. The FFVAs—and needs of the data—were not yet quantitative and were not for predictive or prescriptive decision-making.

A key finding regarding FFVA methods is that the companies were satisfied with the data and method for making “risk-informed” decisions. That said, beyond the basic completion of an FFVA and beginning to set a strategy the companies had very low confidence in the data or methods to make higher level decisions. This method development is a gap.

### Corporate or enterprise-level risk management

#### Formal enterprise-wide assessment systems

This section examined the corporate—rather than quality assurance or food safety—decision-making. Many companies had a corporate-wide risk assessment program (80%), where 11% stated no, and 2% did not know. Regarding a formal COSO/Enterprise Risk Management, 61% identified this as their system, 24% stated no, and 18% did not know.

Expanding on this risk awareness the survey questions addressed the likelihood of food fraud incidents in the next year where most felt the likelihood was medium (29%), very high (16%), high (16%), and low or very low (24%) (Table 5). The major awareness and concern were for “adulterant-substances” and “counterfeiting” where 10% felt an incident was very high in one year, 14% high, 16% low, and 30% very low. Overall, for all types of food fraud, 75% of the respondents felt the chance was low/very low/don’t know.

**Table 5:** Corporate or enterprise-level risk management—likelihood of a food fraud incident in total and by fraud type

Q26: What is the likelihood of any type of food fraud incident occurring at least once to your company in the next 12 months? (You decide on the risk rank)							
	Very High	High	Medium	Low	Very Low	Don’t Know	Prefer not to answer
N ttl	7	11	12	11	20	5	0
%ttl	16	16	29	12	12	9	0
Q27: For the food fraud types listed below, please rank the likelihood of such incidents occurring at least once to your company in the next 12 months?							
Total (%)	Very high	High	Medium	Low	Very low	Don’t Know	
Adulterant-substances	10	14	16	13	30	20	
Tampering	6	9	14	27	29	18	
Theft	9	9	10	23	27	22	
Stolen goods	6	8	6	24	34	22	
Smuggling	1	5	5	23	39	24	
Gray market/diversion	6	10	9	18	30	27	
Counterfeiting/intellectual property rights	5	9	10	18	32	24	

A key finding regarding likelihood is that it is important to understand there is little need to convince the companies that there is a problem but there is a need for more focus on methods to conduct vulnerability assessments and to support resource-allocation decision-making.

#### Food fraud specific systems

This set of questions addressed the corporate processes to manage food fraud. The first question asked if their company had a formal “corporate level” (e.g., not division, company, or group level) food fraud policy and results were that 46% did, 41% did not, 4% did not know.

A key finding regarding a future question is whether the respondents consider their “Food fraud policy” to be stand-alone or included within another policy such as “Food safety” or “Food defense.” Also, a goal was to understand if those that do have a policy only consider adulterant-substances to be in-scope.

The last question in this group asked if they had a “formal food fraud strategy (plan, tactics, and standard operating procedures) at the Corporate or Company-level” which led to a response of that 43% did, 43% did not and 4% did not know. While no further correlation was analyzed, this was the same split as the policy question.

A key finding regarding a future question is to review the relationship between formal policy and the implementation of a formal strategy, and vice versa.

A key finding regarding FFVA management is that with 43% of the respondent companies stating they had a formal corporate level food fraud policy and also a FFPS, this implies a high adoption rate for the new compliance requirements. That said further analysis should be conducted on the breadth and depth of the policies and strategy such as whether they are actually within another policy and if they only cover adulterant-substances.

This preliminary study provided insight on the data collection needs of manufacturers and brand owners.

## Discussion

Addressing food fraud is an emerging and evolving requirement both holistically and specifically to each food type. This data collection survey led to several key conclusions. First, it appears that companies are generally finding “enough” (quantity) and “good enough” (quality) data to conduct their current assessments and meet their compliance needs. It also appears that the early data collection needs are qualitative (lists of incidents) and support descriptive analysis (basic frequencies). Also, the current methods do not require frequent updates since the assessments include compliance requirements to be updated quarterly or annually. Future data collection needs will be defined after government inspectors, or certification auditors have challenged the compliance requirements.

Second, regarding the overall process, the survey respondents demonstrate that there is a need for more guidance or harmonization on vulnerability assessments, strategy development and management, and correlation to all other enterprise-wide risks (ERM/COSO).

Third, from the survey respondents, there is the general activity of conducting food fraud vulnerability assessments, but there is a lack of clarity or direction on the scope (e.g., all types of fraud) and confidence in the conclusions (e.g., the justification or need to respond). This survey suggests there is a need for more definition and formality of the method and process for addressing food fraud.

Finally, the risk assessors in the survey population require more guidance to define their future data collection requirements and needs including a focus on harmonizing terms, vulnerability assessment methods, and then of agreement on a common policy or strategy. Current activities by groups such as Codex Alimentarius and the EU Food Integrity Project will help meet this need.

For further review—beyond reviewing “what is food fraud” or “how to detect fraud”—a simple review of incidents and a scientific discussion about detection methods – companies and countries are now asking “how should we start” and “how much is enough?” This insight includes support in defining the scope and scale of a food fraud policy, prevention strategy, vulnerability assessments, and then, finally, selection and management of countermeasures and control systems. Also, this survey suggests there is a need for more definition and formality of the method and process for addressing food fraud. Specifically, there is a focus on assessing only ingredients and not all products as well as only adulterant-substances and not all types of food fraud.

## Methods

The survey included a distribution list to first a “core expert group” of 50 food fraud experts at 50 companies and then second to a “public” survey that was posted online. The core expert group was selected by the researchers to identify one individual at each manufacturer or brand owner company who was known to be familiar with food fraud prevention. The public online survey was distributed to Michigan State University Food Fraud Initiative List serve contacts including through social media. This research complies with the Michigan State University IRB requirements. The respondents signaled their compliance by accepting the first survey question.

The survey structure includes 49 questions separated into sections:

- Demographics: 13 questions—this used a standard MSU template that allows for meta-analysis
- Food fraud management: 12 questions
- Data availability and needs: 8 questions
- Corporate or enterprise-level risk management:
  - Formal enterprise wide systems: 8 questions
  - Food fraud specific systems: 7 questions
- Food fraud and supply chain trust: 1 question

Previous surveys and research projects were used to create these questions. An internal review and test of the questions were conducted with four researchers. Later, the survey set was piloted by an expert panel, and then finally, the survey was further refined before full distribution. The survey instrument was conducted using the SurveyMonkey Incorporated product.

## Data Availability

The datasets generated during and/or analyzed during the current study are available from the corresponding author on reasonable request.
